# Uncovering the immune-related PCD genes in chronic rhinosinusitis with nasal polyps inflammatory progression: a machine learning and functional validation study

**DOI:** 10.3389/fcell.2026.1702613

**Published:** 2026-03-02

**Authors:** Minliang Chen, Ting Li, Hong Jiao, Quanfa Song

**Affiliations:** Department of Otolaryngology, Sunshine Union Hospital, Weifang, Shandong, China

**Keywords:** chronic rhinosinusitis with nasal polyps, immune cell infiltration, immune-related programmed cell deathgenes, machine learning, prognostic signatures and model, validationanalysis

## Abstract

**Objective:**

This research aimed to explore key immune-related programmed cell death (PCD) genes and associated molecular mechanism on chronic rhinosinusitis with nasal polyps (CRSwNP), which further providing new perspectives for disease prognosis and therapy.

**Method:**

The microarray data were downloaded from the public GEO database. The immune-related PCD genes (co-genes) were identified based on DEGs of CRSwNP vs. normal, immune cells explored by WGCNA as well as PCD genes from published articles, followed by enrichment and PPI network analysis. Important signature genes were screened using machine learning methods, followed by nomogram validation. Then, the immune infiltration, GSEA pathway, target drug and clustering analysis associated with signature genes were further investigated. Finally, validation analysis based on clinical samples were performed to test the expression of signature genes.

**Results:**

A total of 54 co-genes were revealed based on 518 DEGs, 1127 immune genes and 520 PCD genes. By three kinds of machine learning analysis, totally five signature genes including *CD209, CYBB, FPR1, IL2RB* and *TYROBP* were explored, which showed an ideal prognostic value using nomogram investigation. Drug prediction analysis showed Sulfinpyrazone and Azacyclonol exhibited the highest combined scores with five signature genes, which were promising drug candidates for CRSwNP. Immune infiltration and GSEA analysis showed that signature genes were dramatically correlated with immune cells like macrophage and pathways associated with immune. Clustering analysis divided the data into two clusters, which primarily enriched in pathways such as cytokine-cytokine receptor interaction. Then, qRT - PCR, Western blot, and ELISA assays showed that the expression levels of all signature genes were in line with the findings of our bioinformatics analysis. This further validated the reliability of our research outcomes.

**Conclusion:**

*CD209, CYBB, FPR1, IL2RB* and *TYROBP* were valuable immune-related PCD signature genes for clinical prognosis of CRSwNP. In addition, macrophages may play a key role in the chronic inflammation process of CRSwNP via activating immune-associated pathways. These findings may provide compelling evidence for early diagnosis and personalized therapeutic approaches in CRSwNP management.

## 1 Introduction

Chronic rhinosinusitis accompanied by nasal polyps (CRSwNP) represents a prevalent chronic inflammatory disorder affecting the upper respiratory tract (Bandi et al., 2023). As the disease progresses, it leads to structural damage and loss of function within the nasal cavity. Due to the persistent and difficult to cure symptoms of CRSwNP, patients often require long-term medication such as nasal steroids, antihistamines, and antibiotics. In some cases, functional sinus surgery (such as functional endoscopic sinus surgery, FESS) may be a necessary treatment option (Baird et al., 2024). However, even with surgical treatment, some patients may still experience recurrence, leading to long-term medical costs and psychological burden (Levi et al., 2024). Thus, understanding the exact pathogenesis of CRSwNP has important clinical significance of early diagnosis, intervention, and treatment strategies for patients with CRSwNP.

Recent studies have increasingly indicated that the development of CRSwNP is not solely driven by traditional allergic reactions or bacterial infections, but involves complex immune responses, cell death mechanisms, and gene regulatory networks (Soliai et al., 2023; Fan et al., 2024). The immune system’s role in CRSwNP has gained significant attention, especially the infiltration and activation of immune cells, which have been shown to be closely associated with the persistence of chronic inflammation (Li et al., 2022). Studies have found that CRSwNP patients exhibit a significant increase in eosinophils, mast cells, T cells, and other immune cells in the nasal polyp tissues, and the overactivation of these cells can exacerbate chronic inflammation and promote disease progression (Ye et al., 2023). Moreover, cell death, as a crucial biological process, has attracted considerable attention in recent years, especially regarding programmed cell death (PCD) related genes, which play an important role in CRSwNP. Diverse forms of programmed cell death, including apoptosis, necrosis, and pyroptosis, could be pivotal in disrupting immune regulation and causing tissue damage in CRSwNP (Morawska-Kochman et al., 2022). However, the immune-related cell death mechanisms in CRSwNP have not been thoroughly explored, particularly the interactions between various immune cell types and their gene signatures during disease progression remain unclear. Recent immune-endotyping research has further characterized gene expression heterogeneity among CRSwNP subtypes, particularly in AERD and non-AERD patients, underscoring diverse immune activation pathways (Nazari et al., 2024). Fortunately, with the development of high-throughput genomics technologies, multi-omics analyses based on big data provide new insights and tools for studying complex immune pathological processes. Gene expression profiling, immune cell infiltration analysis, and WGCNA have been applied in CRSwNP research, revealing potential key genes and their regulatory networks (Zhu et al., 2022; Wang H. et al., 2024). These technologies not only help to deepen the understanding of the immunological basis of CRSwNP but also provide new approaches for early diagnosis, precise treatment, and individualized drug development.

In this study, we aimed to use machine learning methods, combined with analysis of immune cell infiltration and analysis of the gene co-expression network, to identify immune-related cell death signatures closely associated with the progression of CRSwNP. Multiple publicly available datasets from the GEO database were utilized to explore the gene expression differences between CRSwNP patients and normal controls, with a particular focus on the characteristics of immune cell infiltration and their relationship with immune-related cell death genes. Various machine learning algorithms were employed to further identify potential biomarkers, which were validated using independent datasets, with the aim of providing new molecular targets for the early diagnosis and treatment of CRSwNP. The findings of this study were expected to not only provided new insights into the immunopathological mechanisms of CRSwNP, but also offered theoretical support for its clinical management. The flowchar for current study was showed in Supplementary Figure S1.

## 2 Materials and methods

### 2.1 Microarray data and data preprocessing

Three datasets including GSE136825 (42 CRSwNP samples and 28 normal samples, platform: GPL20301), GSE17926 (17 CRSwNP samples and 7 normal samples, platform: GPL24676), GSE72713 (6 CRSwNP samples and 3 normal samples, platform: GPL570), were download from Gene Expression Omnibus (GEO) database as the training datas. In addition, two datasets including GSE23552 (11 CRSwNP samples and 13 normal samples, platform: GPL5175) and GSE36830 (6 CRSwNP samples and 6 normal samples, platform: GPL570) were used as validation datasets. The batch effects were removed from the three training datasets, and the resulting data were combined to form the training datasets using sva package (version: 3.50.0) in R (Leek et al., 2012). By utilizing the probe expression matrix and the annotation file, those probes which had no correspondence with a gene symbol were eliminated. In the case of genes that had multiple corresponding probes, the mean expression value of these probes was computed and employed as the expression value for that specific gene.

### 2.2 Immune cell expression analysis

Immune cell expression levels were calculated using two independent algorithms. Briefly, the infiltration of 28 immune cells was assessed based on gene expression data from the training set, utilizing the ssGSEA algorithm in R. Scores for 22 immune cells were derived using the CIBERSORT algorithm. Differences in immune cell distributions between CRSwNP and normal samples were tested using the Wilcoxon test. Immune cell types showing significant and consistent differences (in the same direction) across both algorithms were selected as optimal immune cells for following analysis.

### 2.3 Differentially expression and enrichment analysis

The limma package in R was used to explore the differentially expressed genes (DEGs) between CRSwNP samples and normal samples from three training datasets. In short, significance analysis of the expression of all genes was carried out based on the log2 fold change (FC) and *P* value. The criteria for selecting DEGs were a Benjamini - Hochberg (BH) - adjusted (adj) *P* < 0.05 and |log_2_FC| > 1. The results were presented visually through a volcano plot and a heatmap. Furthermore, GO function and KEGG pathway analyses were conducted on the DE-RBRGs using the clusterProfiler package in R. The GO function encompasses biological process (BP), cellular component (CC), and molecular function (MF). BH-adj *P* < 0.05 was set as the threshold for the current enrichment analysis.

### 2.4 WGCNA analysis and model gene enrichment investigation

The analysis started by performing a variance analysis on the expression matrix of OS samples to identify the top 5000 genes with the highest variability across the samples. Next, WGCNA (version 1.72-5) (Langfelder and Horvath, 2008) was applied to detect gene modules with significant co-expression patterns. At the beginning, the soft thresholding approach was employed to transform the adjacency matrix into a continuous scale ranging from 1 to 20. This was done to ensure that the resulting network conformed to a power-law distribution, which can more precisely represent the characteristics of biological networks. In the present study, a soft threshold value of 0.85 was selected for constructing the network, since it was the first value that satisfied the necessary criteria (minModuleSize = 50). Subsequently, the blockwiseModules function was utilized to generate a scale-free network, and an analysis of module partitioning was carried out. For every module, the module membership and gene significance were computed. In addition, the GO function and KEGG pathway enrichment analysis were executed on the genes of the selected modules.

### 2.5 The co-genes investigation and interaction analysis

The PCD related genes were obtained from the previous published articles (Qin et al., 2023; Zhou et al., 2023). The common genes (co-genes) among DEGs, module genes and PCD related genes were explored by using VENN plot analysis. Moreover, based on the STING database (version: 11.0) (Szklarczyk et al., 2016), the protein interaction information was retrieved, and the protein-protein interaction (PPI) pairs among the DE-RBRGs were predicted with a combined_score greater than 0.4. The PPI network was established using the Cytoscape (version: 3.7.1) software. Additionally, the Maximum Clique Centrality (MCC), Maximum Neighborhood Component (MNC), Degree Centrality (Degree), and Edge Percolated Component (EPC) topology algorithms within the cytoHubba package of the Cytoscape software were applied to identify hub genes. This identification was based on the TOP 30 nodes in the PPI network.

### 2.6 Signature genes investigation

Three machine-learning algorithms were utilized to recognize signature genes for CRSwNP. First, LASSO, as a regularization regression method, employs an L1 penalty to achieve feature sparsity, making it suitable for dimensionality reduction in high-dimensional data. The optimal penalty parameter λ was determined through 10-fold cross-validation (cv.glmnet), with the data standardized (standardize = TRUE) and the model configured for binary classification (family = “binomial”). Secondly, Support Vector Machine - Recursive Feature Elimination (SVM-RFE) is a recursive feature elimination algorithm based on support vector machines. It employs a linear kernel function to ensure the interpretability of feature weights. The penalty parameter C is optimized through 10-fold cross-validation and grid search, while the proportion of features removed per iteration (1%–10%) is set to enhance selection robustness. Random Forest (RF) evaluates feature importance by constructing multiple decision trees (ntree = 500–2000). The number of randomly selected features per tree (mtry) was optimized using 10-fold cross-validation via the tuneRF function. A minimum node size (nodesize = 10) was set to control model complexity. Features were ultimately selected based on significant reductions in either the Mean Decrease in Accuracy or the Gini index (Mean Decrease Gini >2). LASSO regression, SVM-RFE, and RF analyses were conducted using the R packages “glmnet version 4.1-8″, “e1071 version 1.7-14″, and “randomForest version 4.7-1.1″, respectively. The intersecting genes identified by these three methods were considered pivotal feature genes for the diagnosis of CRSwNP.

### 2.7 Diagnostic evaluation

We applied the Wilcoxon signed rank test to assess the differentially expression for signature genes between CRSwNP group and normal group based on all training datasets and validation datasets. Then, by using the pROC package (version 1.12.1) (Robin et al., 2011), the Receiver Operating Characteristic (ROC) curve analysis was performed to determine the Area Under the Curve (AUC) value for each signature gene in CRSwNP versus control samples. Subsequently, the signature genes were used to construct a nomogram through the rms package in R. With the rms package (version: 6.3-0) (Ye et al., 2019), a nomogram was created according to the nomoScore values of all genes. Moreover, a calibration curve, a decision curve, and a clinical curve analysis were carried out to assess the performance of the nomogram.

### 2.8 Immune landscape, target drugs, and GSEA analysis based on signature genes

The Pearson correlation coefficient, with the help of the ggplot2 (version: 3.5.0) package in R, was used to analyze the correlation between feature genes and immune cells. The pheatmap was employed to visualize the results. In addition, drugs that target feature genes were identified by referring to the Drug Signatures Database (DSigDB) (Yoo et al., 2015). Subsequently, Cytoscape software was utilized to uncover the target drugs of signature genes. Furthermore, Gene Set Enrichment Analysis (GSEA) (Reimand et al., 2019) was carried out on the feature genes to find significant pathways. For this analysis, the thresholds were set as *P* < 0.05 and NES >1.

### 2.9 Clustering analysis

To investigate different clusters of CRSwNP, the ConsensusClusterPlus package (Wilkerson and Hayes, 2010) in R software was used for analysis, followed by the Principal Component Analysis (PCA) to further validate the stability of clustering results. Subsequently, the t-test was used to analyze the correlation between clusters and clinical information. Finally, with |NES| > 1 and BH-adj *P* < 0.05, the GSEA analysis was performed to identify the KEGG pathways enriched by different clusters.

### 2.10 Patient recruitment and sample collection

The diagnosis of CRSwNP was made in accordance with the clinical diagnostic criteria stipulated in the International Consensus Statement on Allergy and Rhinology (Orlandi et al., 2021). The criteria for exclusion encompassed ciliary dysfunction, autoimmune diseases, cystic fibrosis, immunodeficiency conditions, malignancies, gastroesophageal reflux disease, chronic rheumatic diseases, and any other disorder that necessitated long-term corticosteroid treatment. The study adheres to the Helsinki Declaration. Nasal polyp tissues were collected from 40 CRSwNP patients who underwent functional endoscopic sinus surgery (FESS) at our hospital between January 2024 and December 2024. Control samples of normal nasal mucosa were obtained from 25 patients undergoing septoplasty without any inflammatory conditions. Each tissue sample was separated into three parts. One part was promptly fixed in a 4% paraformaldehyde solution for the purpose of histological analysis. Another part was preserved in RNAlater solution (Thermo Fisher Scientific, AM7020) at a temperature of −80 °C for the extraction of RNA. The remaining part was rapidly frozen in liquid nitrogen for the extraction of proteins. This research project had received approval from the institutional ethics committee. Additionally, written informed consent was duly acquired from every participant involved in the study.

### 2.11 Peripheral blood PBMC isolation, macrophage differentiation, and treatment

Peripheral blood (5 mL) was collected from CRSwNP patients (n = 15) and healthy controls (n = 10). Human peripheral blood mononuclear cells (PBMCs) were isolated using Ficoll density gradient centrifugation and enriched by adherent culture. PBMCs were seeded into RPMI 1640 medium containing 10% FBS at a density of 1 × 10^6^ cells/mL per well. After incubation for 2 h at 37 °C under 5% CO_2_, non-adherent lymphocytes were removed by washing. The adherent cells were primarily monocytes. Subsequently, 50 ng/mL of macrophage colony-stimulating factor (M-CSF, PeproTech, USA) was added to the culture system, and the cells were cultured for 5–7 days at 37 °C under 5% CO_2_. The culture medium was refreshed every 3 days to induce the differentiation of PBMCs into M0 macrophages. Differentiation was confirmed by microscopic observation, which revealed cells with adherent, spindle-shaped, or irregular morphologies. Cells were transfected with siRNA-NC, FPR1 siRNA, empty vector (Vector), or TYROBP overexpression plasmid (TYROBP-OE) using Lipofectamine 3000 reagent (Invitrogen, USA) in CRSwNP-derived macrophages (CRSwNP-Mφ). After transfection for 24 h, except for the healthy control-derived macrophages (HC-Mφ) and the CRSwNP-Mφ groups under basal expression detection, all other groups were switched to fresh culture medium containing 100 ng/mL lipopolysaccharide (LPS, Sigma-Aldrich) to simulate an inflammatory microenvironment and further stimulated for 24 h. Upon stimulation completion, cell pellets and culture supernatants were collected separately for subsequent RNA, protein, and cytokine detection.

### 2.12 RNA extraction and quantitative real-time PCR

Total RNA was isolated using TRIzol reagent (Invitrogen, 15596026) following the manufacturer’s guidelines. The concentration and quality of the RNA were evaluated using a NanoDrop 2000 spectrophotometer (Thermo Fisher Scientific) and an Agilent 2100 Bioanalyzer (Agilent Technologies). Only those samples with an RNA integrity number (RIN) greater than 7.0 were employed for subsequent analysis. First - strand cDNA was generated from 1 μg of total RNA with the PrimeScript RT reagent Kit (Takara, RR037A). The qRT-PCR was carried out using TB Green Premix Ex Taq II (Takara, RR820A) on an ABI 7500 Real - Time PCR System (Applied Biosystems). GAPDH served as an internal control. The primers utilized are presented in Table 1. The PCR program consisted of an initial denaturation at 95 °C for 5 min, followed by 35 cycles of denaturation at 95 °C for 30 s and annealing at 52 °C for 30 s. The relative expression levels were calculated using the 2^−ΔΔCT^ method (Livak KJ, 2001).

**TABLE 1:** The detail information for all primers used in current study.

Primer	Sequence(5′-3′)
CD209-F	ACT​CCA​AGG​AAC​CAA​GAC​TGC
CD209-R	GCG​TCT​TGC​CTG​GAT​TGT​TC
CYBB-F	GGG​AAC​TGG​GCT​GTG​AAT​GA
CYBB-R	CCA​GTG​CTG​ACC​CAA​GAA​GT
FPR1-F	CTG​ATC​GCC​CTC​ATT​GCT​CT
FPR1-R	CAG​GGC​CCA​ATG​ATC​ACC​TT
IL2RB-F	GAT​TTT​CAG​CCA​CCC​CCT​GA
IL2RB-R	CTG​GAC​CAG​GGG​AAA​CTG​AC
TYROBP-F	CGACCCGGAAACAGCGT
TYROBP-R	GAG​GTC​GCT​GTA​GAC​ATC​CG
GAPDH-F	TCA​AGG​CTG​AGA​ACG​GGA​AG
GAPDH-R	TGG​ACT​CCA​CGA​CGT​ACT​CA

GAPDH, Glyceraldehyde 3-phosphate dehydrogenase.

### 2.13 Western blot analysis

Total protein was isolated using RIPA lysis buffer (Beyotime, P0013B) supplemented with a protease inhibitor cocktail (Roche, 04693159001). The protein concentration was measured using the BCA protein assay kit (Thermo Fisher Scientific, 23225). Equal quantities of protein (30 μg) were subjected to separation via 10% SDS-PAGE and then transferred onto PVDF membranes (Millipore, IPVH00010). The membranes were blocked with 5% non-fat milk and then incubated with primary antibodies at 4 °C overnight. The primary antibodies used were as follows: anti-CD209 (Abcam, ab259434, dilution 1:1000), anti-CYBB (Cell Signaling Technology, #80897, dilution 1:1000), anti-FPR1 (Proteintech, 13802-1-AP, dilution 1:800), anti-IL2RB (R&D Systems, MAB224, dilution 1:1000), anti-TYROBP (Santa Cruz, sc-20783, dilution 1:800), anti-iNOS (Abcam, ab15323, dilution 1:1000), anti-Arg-1 (Cell Signaling Technology, #93668, dilution 1:200), anti-p38 (Cell Signaling Technology, #8690S, dilution 1:1000), anti-phospho-ERK1/2 (Cell Signaling Technology, #9101S, dilution 1:1000), anti-ERK (Santa Cruz, sc-93, dilution 1:1000), anti-phospho-p38 (Merck Millipore, 09-272, dilution 1:1000), and anti-GAPDH (Cell Signaling Technology, #5174, dilution 1:2000). After washing, the membranes were incubated with HRP-conjugated secondary antibodies and visualized using the ECL detection system (Bio-Rad, 1705061).

### 2.14 ELISA analysis

To further investigate the functional roles of signature genes in CRSwNP, the levels of expression of crucial inflammatory cytokines and chemokines were determined by means of ELISA. Human eosinophilic cells (EoL-1) were transfected with specific siRNA targeting FRP1 (purchased from Thermo Fisher Scientific, USA) and an overexpression plasmid targeting TYROBP (purchased from OriGene, USA). After 24 h of transfection, the cells were stimulated with lipopolysaccharide (LPS, 100 ng/mL) for 24 h to enhance cytokine production. Then, cell culture supernatants were collected and centrifuged to remove debris. The concentrations of IL-8, CCL5, TNF-α and IL-6 in the supernatants were quantified using commercial ELISA kits (R&D Systems, USA) according to the manufacturer’s instructions. In brief, the standards and the samples were placed into the pre-coated wells and allowed to incubate for 2 h at ambient temperature. Following the washing step, the detection antibodies were added. Subsequently, the streptavidin-HRP conjugate and the substrate solution were introduced. Once the reaction was terminated, the absorbance was gauged at a wavelength of 450 nm with the aid of a microplate reader (BioTek Synergy H1, USA). All results were visualized using bar chart showing mean ± SD.

### 2.15 Statistical analysis

Statistical analyses were carried out with GraphPad Prism 9.0 software. A P-value less than 0.05 was regarded as statistically significant. To assess the differences between two groups, either the unpaired t-test or the Wilcoxon Rank-Sum Test was employed. Pearson’s correlation was used to evaluate the relationships among variables. The relative expression levels of mRNA and protein were standardized against those of the control groups.

## 3 Results

### 3.1 The expression analysis of immune cells

**FIGURE 1:**
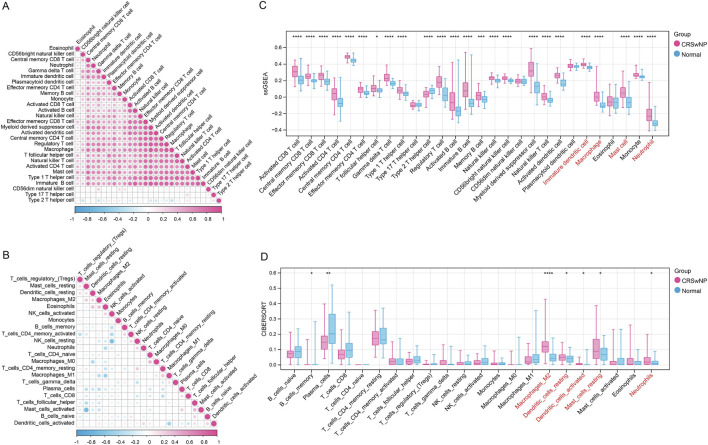
The immune cell expression between chronic rhinosinusitis with nasal polyps (CRSwNP) samples and normal samples. **(A)** The correlation of 28 kinds of immune cells based on ssGSEA analysis. **(B)** The immune cell expression bet the correlation of 22 kinds of immune cells based on CIBERSORT analysis. **(C,D)** The significant immune cells that differentially expressed between CRSwNP samples and normal samples based on ssGSEA and CIBERSORT analysis, respectively. *, *P* < 0.05; **, *P* < 0.01; ***, *P* < 0.001; ****, *P* < 0.0001.

After data preprocessing, a total 65 CRSwNP samples and 38 normal samples were revealed based on three training datasets (GSE136825, GSE179265 and GSE72713). The PCA results revealed that prior to batch correction, samples clustered according to their respective datasets, whereas after batch correction, the distribution of samples in the principal component space became more aggregated, with a significant reduction in inter-batch differences. This indicates that batch effects were effectively removed, making the data more suitable for subsequent analyses (Supplementary Figure S2). Based on the expression profile data from the training set, the levels of 28 and 22 types of immune cells were obtained using the ssGSEA algorithm and the CIBERSORT algorithm, respectively. The results revealed significant correlations among different immune cells (Figures 1A,B). Subsequently, both algorithms identified significant differences in Immature dendritic cell, Macrophage, Mast cell, and Neutrophil between CRSwNP and normal groups, suggesting these cells may play a critical role in the CRSwNP microenvironment (all *P* < 0.05, Figures 1C,D).

### 3.2 Differentially expressed genes (DEGs) investigation

**FIGURE 2:**
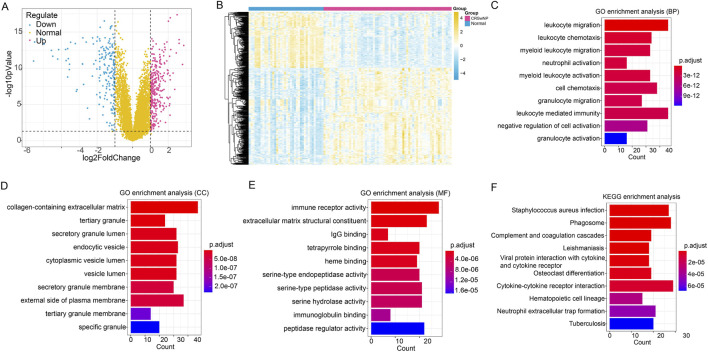
The results of differentially expressed genes (DEGs) investigation. **(A)** The volcano plot showed the DEGs between CRSwNP samples and normal samples. **(B)** The heatmap showed all samples could be separated by different groups (samples). **(C–E)** The TOP 10 significant GO-BP, GO-CC and GO-MF functions assembled by DEGs. **(F)**, the TOP 10 significant KEGG pathways enriched by DEGs.

In total, 518 DEGs were identified between CRSwNP samples and normal samples using |log2FC|>1 and p < 0.05 as cutoffs, among which 329 genes were upregulated and 189 genes were downregulated (Figure 2A). The heatmap analysis shows that all samples are clearly divided into two groups, with CRSwNP samples and normal samples completely separated (Figure 2B). GO function analysis revealed that these DEGs were mainly enriched in BP like leukocyte migration (GO:0050900), CC such as collagen-containing extracellular matrix (GO:0062023), and MF including immune receptor activity (GO:0140375) (Figures 2C–E). Additionally, KEGG pathway analysis showed that the DEGs were significantly enriched in *staphylococcus aureus* infection (hsa05150) (Figure 2F), suggesting a potential role of bacterial infection in the pathogenesis of CRSwNP.

### 3.3 WGCNA analysis

**FIGURE 3:**
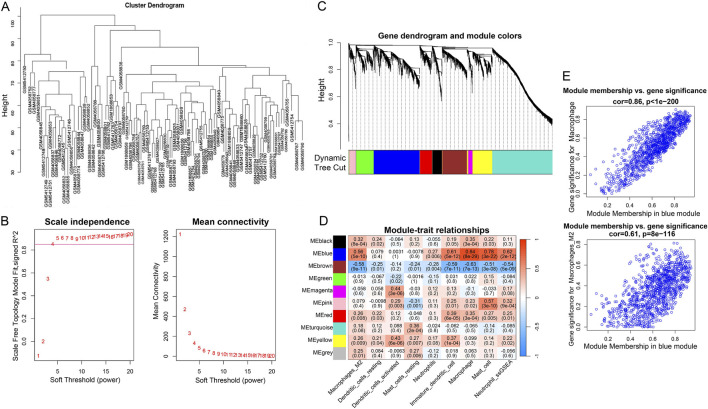
The result of WCGNA analysis. **(A)** The hierarchical clustering analysis based on the TOP5000 genes. **(B)** The scale free soft-threshold distribution. **(C)** Clustering analysis for models: dynamic tree cut and merged dynamic represented the module before and after the merge module; the different colors in the Figure represented different modules. **(D)** The heatmap for correlation between modules and immune cells. **(E)** The gene significant analysis between modules and Macrophage: blue represented negatively correlation; the darker the color, the stronger the correlation.

The hierarchical clustering results based on the TOP5000 genes demonstrated good data quality and reliability. Samples were tightly clustered within their respective groups and well - separated between groups, providing a solid foundation for subsequent analysis (Figure 3A). Then, WGCNA analysis was conducted. The soft-threshold of 4 was chosen based on the scale - free topology fit index and power - law relationship tests, and a fitting degree of 0.85 was set to ensure the reliability of the network construction (Figure 3B). The adjacency matrix was constructed using the soft thresholding method, followed by the calculation of the gene topological matrix, derivation of the inter-gene dissimilarity coefficient, and generation of a systematic clustering tree. The dynamic tree cutting algorithm was then employed for module division, with similar modules merged and a minimum module gene count of 50. Ultimately, nine gene modules were obtained (excluding the gray module) (Figure 3C). The correlation between these modules and immune cells was visualized with a heatmap (Figure 3D). The analysis revealed that the blue module (r = 0.84, *P* < 0.05, 1127 genes) had the strongest positive correlations with Macrophage, identifying them as key module in this study. The gene significant analysis for Macrophage showed a strong correlation among module genes and Macrophage (Figure 3E). Thus, totally 1127 module genes in blue module were enrolled for the following analysis.

### 3.4 The comprehensive analysis based on module genes

**FIGURE 4:**
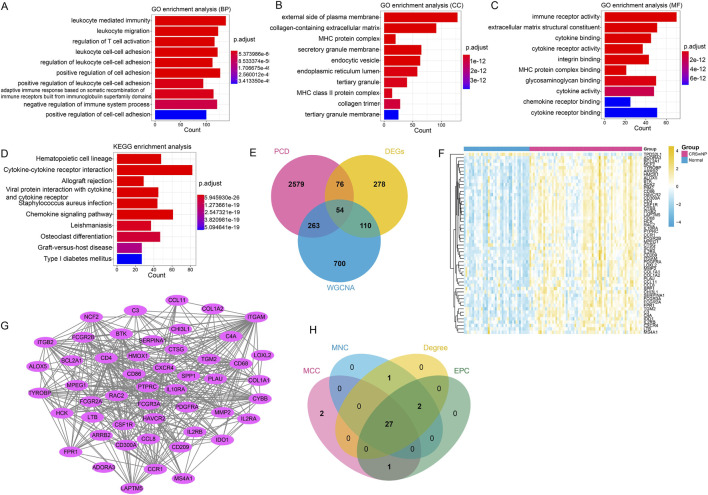
The comprehensive analysis based on module genes. **(A–C)** The TOP 10 significant GO-BP, GO-CC and GO-MF functions assembled by module genes, respectively. **(D)** The TOP 10 significant KEGG pathways enriched by module genes. **(E)** The VENN plot revealed 54 common genes (co-genes) related with Macrophage based on DEGs, module genes and programmed cell death (PCD) related genes. **(F)** The heatmap analysis for all co-genes. **(G)** A PPI network constructed with co-genes: the node represented different genes, while the line between two node represented interaction. **(H)** The VENN plot revealed 27 hub genes by using four topology algorithms.

The enrichment analysis for 1127 module genes showed that these module genes were mainly assembled in functions including leukocyte mediated immunity (BP, GO:0002443) (Figure 4A), external side of plasma membrane (CC, GO:0009897) (Figure 4B) and immune receptor activity (MF, GO:0140375) (Figure 4C). In addition, these module genes were predominantly enriched in pathways such as cytokine-cytokine receptor interaction (hsa04060) (Figure 4D). Then, the VENN plot analysis revealed totally 54 co-genes that associated with Macrophage based on 518 DEGs, 1127 module genes and 520 published PCD genes (Figure 4E). The heatmap analysis showed that most of these co-genes were upregulated in CRSwNP samples, but downregulated in normal samples (Figure 4F). Additionally, a PPI network was established on the basis of these co-genes (Figure 4G). The result showed that there were 50 nodes and 406 interactions in current PPI network. In addition, the topology analysis based on MCC, MNC, EPC and Degree algorithms revealed 27 hub genes from TOP 30 nodes in PPI network (Figure 4H).

### 3.5 Signature genes investigation

**FIGURE 5:**
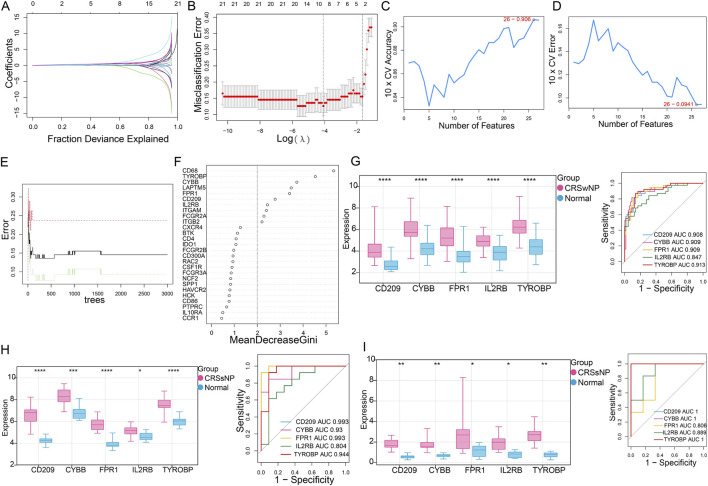
The feature genes investigated by three machine learning methods. **(A,B)**, LASSO Cox analysis revealed nine optimal genes: the Y-axis in the **(A)** represented the coefficient of the variable, while the X-axis represented the value of log(lambda); the two dotted lines in **(B)** Represented two special lambda values: lambda. min on the left and lambda.1se on the right; the lambda between these two values was considered appropriate. **(C)** The accuracy for SVM-RFE analysis. **(D)** The error rate for SVM-RFE analysis. **(E)** Relationship between the number of trees and the error rate in a RF model. **(F)** The Top 10 genes selected by using RF algorithm. **(G)** The validation analysis for signature genes based on training dataset: the left box plot showed the expression of signature genes between CRSwNP group and normal group, while the right ORC curve represented the AUC value for all signature genes. **(H–I)** The validation analysis for signature genes based on two validation dataset GSE23552 and GSE36830, respectively. *, *P* < 0.05; **, *P* < 0.01; ***, *P* < 0.001; ****, *P* < 0.0001.

Advanced machine learning algorithms (LASSO, SVM-RFE, and Random Forest) were employed to analyze the 27 hub genes. For the LASSO algorithm, the minimum standard with higher accuracy was selected through ten-fold cross-validation to construct the LASSO classifier, identifying a total of 9 signature genes (Figures 5A,B). For the SVM-RFE algorithm, the classifier error is minimized when the number of features is 26. Therefore, SVM-RFE has identified 26 signature genes (Figures 5C,D). Moreover, the Random Forest algorithm uncovered the top 10 genes, with the criterion being a MeanDecreaseGini value exceeding 2 (Figures 5E,F). The intersection of genes selected by these various methods resulted in a final set of five feature genes including *CD209*, *CYBB*, *FPR1*, *IL2RB* and *TYROBP*, which were deemed signature genes for investigation into CRSwNP (Supplementary Figure S3). Then, the evaluation analysis was performed on five signature genes. The result showed that all these five genes were significant overexpressed in CRSsNP group than those in normal group either in training dataset (Figure 5G) or in two validation datasets (all *P* < 0.05) (Figures 5H,I). In addition, the result of ROC analysis showed that AUC values for each gene was larger than 0.804 in both training dataset (Figure 5G) and validation dataset (Figures 5H,I), indicating a very good diagnostic value for all signature genes.

### 3.6 Diagnostic evaluation and immune correlation analysis for signature genes

**FIGURE 6:**
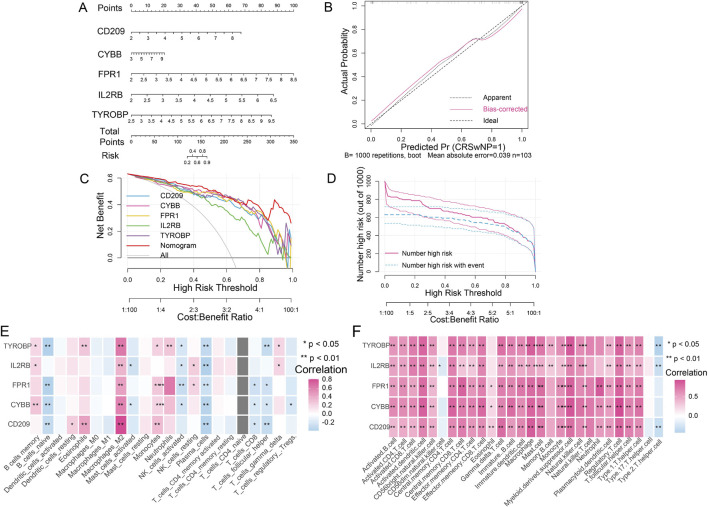
Diagnostic evaluation and immune correlation analysis for signature genes. **(A)** Nomogram model constructed by five signature genes predicting the risk of CRSwNP: the nomogram was used by summing all points identified on the scale for each variable; the total points projected on the bottom scales indicate the risk of CRSwNP. **(B)** Calibration curve analysis to validate the predicable of nomogram. **(C)** The decision curve analysis used to evaluate optimal threshold for current nomogram. **(D)** The clinical curve for evaluating the predictive power of nomograph model. **(E)** The correlation between signature genes and 28 kinds of immune cells. **(F)** The correlation between signature genes and 22 kinds of immune cells. *, *P* < 0.05; **, *P* < 0.01.

A nomogram was established using grouping information and expression of signature genes (Figure 6A). Specific scores were assigned to each variable on the scoring axis for quantitative evaluation. In the nomogram, the contribution of different genes (CD209, CYBB, FPR1, IL2RB, TYROBP) to the prediction of CRSwNP risk was compared. The scores of each gene on the scoring axis reflect their relative importance in risk prediction. For example, genes with a wider score range may have a greater impact on risk assessment. Then, the calibration curve was used to evaluate the predictive performance of the nomogram. The calibration curve demonstrated minimal discrepancy between the actual and predicted risks of CRSwNP disease occurrence, indicating high predictive accuracy of the nomogram for CRSwNP (Figure 6B). Decision curve analysis (DCA) demonstrated that the nomogram curve was superior to the gray line curve, indicating better clinical benefits for patients using the nomogram (Figure 6C). To visually evaluate the clinical efficacy of the nomogram, a clinical impact curve was plotted based on the DCA curve. The “Number high risk” curve closely approximated the “Number high risk with event” curve when the high-risk threshold ranged from 0.7 to 1, indicating the nomogram’s ideal predictive performance (Figure 6D). Moreover, the immune correlation analysis between signature genes and immune cells showed that all five signature genes were significantly correlated with Macrophage both by using ssGSEA (Figure 6E) and CIBERSORT (Figure 6F) analysis (all *P* < 0.05).

### 3.7 Drug prediction and GSEA analysis

The drug prediction on five signature genes was performed by using DSigDB database. The TOP 10 signature gene-related drugs were shown in Supplementary Figure S4A. The most significant interactions include Sulfinpyrazone (TTD 00011126), Azacyclonol (HL60 UP), and Superoxide (BOSS), which exhibited the highest combined scores. Additionally, compounds such as Pyrrolidine dithiocarbamate (CTD 00001021) and Ionomycin (CTD 00007090) showed notable interactions with adjusted p-values of 0.03 and 0.02, respectively. Moreover, the GSEA analysis was performed on all five signature genes. The result showed that *CD209*, *CYBB*, *FPR1*, *IL2RB* and *TYROBP* were mainly enriched in pathway like autoimmune thyroid disease (Supplementary Figures S4B–F).

### 3.8 Clustering investigation

Unsupervised clustering analysis of CRSwNP samples was performed based on five characteristic genes. As shown in Supplementary Figure S5A,B, the increasing trend of the cumulative distribution function (CDF) values relative to the consensus index indicates appropriate classification. According to the CDF curve and Delta area, when the clustering index “k” increases from 2 to 9, k = 2 was proven to be the optimal point for achieving the greatest inter-cluster differences (with relatively stable curves). A total of two distinct clusters (C1 and C2) were obtained, containing 25 and 40 CRSwNP samples, respectively (Supplementary Figure S5C). The PAC further validated the stability of the clustering results, with the PAC minimum (optimal K) remaining at 2 (Supplementary Figure S5D). Then, the PCA analysis showed that all genes could be separated by different clusters (Supplementary Figure S5E). The box plot analysis for five signature genes revealed that they were dramatically different expressed between cluster 1 and cluster 2 (all *P* < 0.01) (Supplementary Figure S5F). Furthermore, the significant KEGG pathways differentially expressed between clusters was investigated by using GSEA analysis. The result showed that totally 132 KEGG pathways such as apoptosis chemokine signaling pathway and cytokine-cytokine receptor interaction were dyregulation between two clusters. The TOP 6 upregulated and TOP 6 downregulated pathways was showed in Supplementary Figure S5G,H, respectively.

### 3.9 Hub genes expression in CRSwNP tissues

**FIGURE 7:**
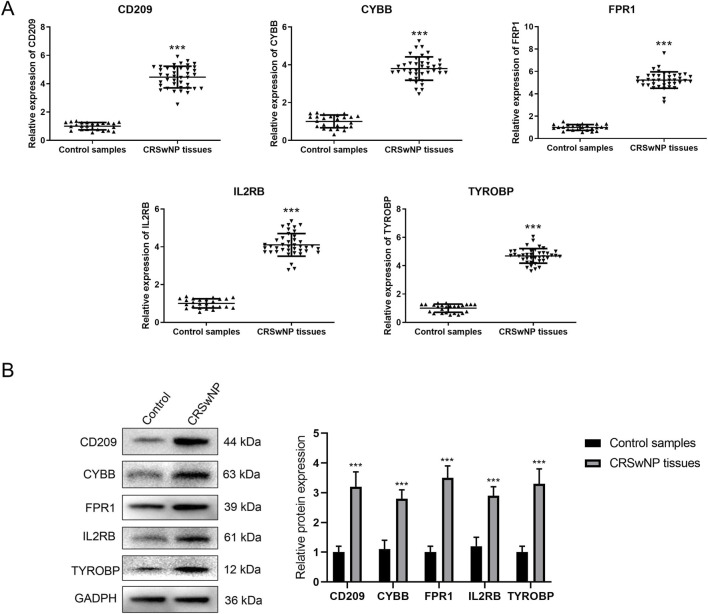
The validation analysis based on clinical samples. **(A)** The results of qRT-PCR analysis for signature genes in CRSwNP group and control group: the X-axis represented different signature genes, while the Y-axis represented the relative expression value of mRNAs. **(B)** The results of Western blot analysis for signature genes in CRSwNP group and control group: the X-axis represented different signature genes, while the Y-axis represented the relative expression value of protein. ***, *P* < 0.001.

To validate the expression patterns of the identified hub genes, we performed qRT-PCR and Western blot analyses on nasal tissue samples from 40 CRSwNP patients and 25 control subjects. The qRT-PCR results showed significantly increased mRNA expression levels of all five hub genes in CRSwNP tissues compared to control tissues (all *P* < 0.001) (Figure 7A). Consistent with the mRNA expression patterns, Western blot analysis revealed significantly elevated protein levels of all five hub genes in CRSwNP tissues compared to control tissues (all *P* < 0.001) (Figure 7B).

### 3.10 The functional role of FPR1 and TYROBP in CRSwNP

**FIGURE 8:**
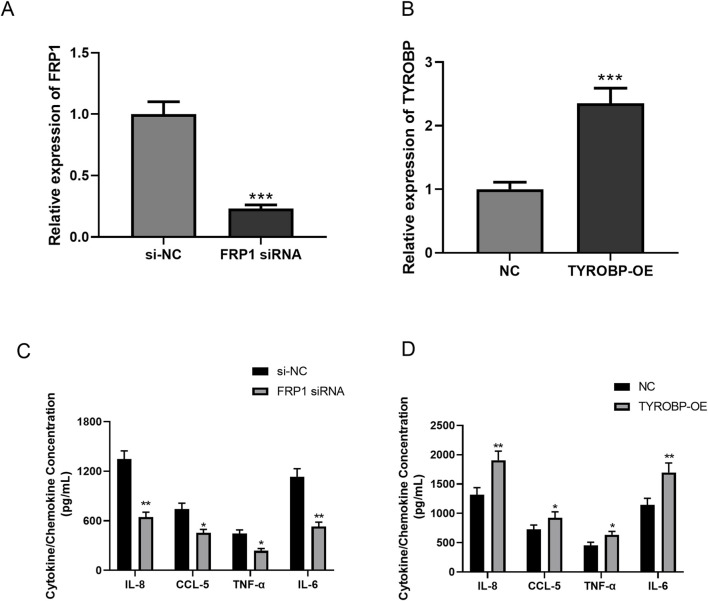
ELISA detection of secretion changes of inflammatory cytokines and chemokines after FPR knockout and TYROBP overexpression. **(A,B)** QRT-PCR detection of transfection efficiency of FPR siRNA **(A)** and TYROBP-OE **(B)**. **(C,D)** The results of ELISA assay in CRSwNP group and control group: the X-axis represented siRNA knockdown **(C)** or overexpression **(D)** of signature genes, while the Y-axis represented the cytokine/chemokine concentration. *, *P* < 0.05; ****, *P* < 0.0001.

QRT-PCR analysis showed that FPR1 siRNA and TYROBP-OE were successfully transfected into EoL-1 cells (both *P* < 0.001, Figures 8A,B). The ELISA results demonstrated that FPR1 knockdown significantly reduced the concentrations of IL-8, CCL5, TNF-α, and IL-6 (*P* < 0.05, Figure 8C), suggesting a role for FPR1 in promoting chemokine production and immune cell recruitment. Conversely, TYROBP overexpression significantly increased the levels of IL-8, CCL5, TNF-α, and IL-6 (*P* < 0.05, Figure 8D), indicating that TYROBP enhances pro-inflammatory responses. The results suggested that *FPR1*, and *TYROBP* may regulate inflammatory pathways in CRSwNP, potentially contributing to disease progression.

### 3.11 Functional validation of FPR1 and TYROBP in peripheral blood immune cells

**FIGURE 9:**
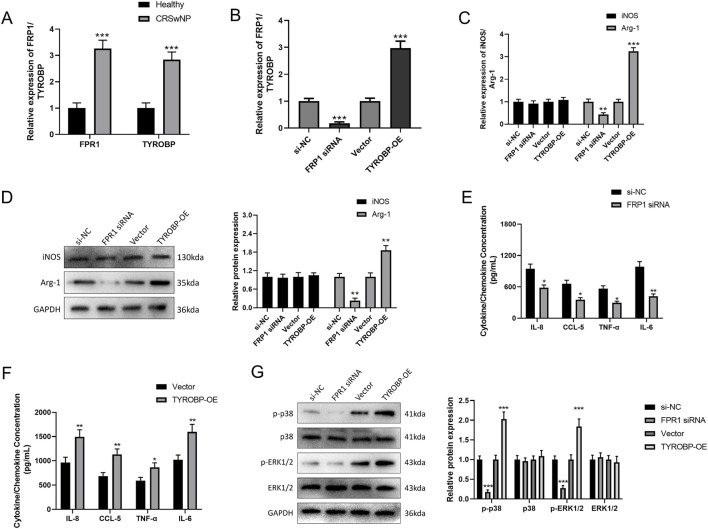
Functional validation of FPR1 and TYROBP in peripheral blood immune cells. **(A)** qRT-PCR analysis of FPR1 and TYROBP mRNA expression in peripheral blood macrophages from CRSwNP patients versus healthy controls. **(B)** qRT-PCR validation of FPR1 siRNA knockdown efficiency and TYROBP overexpression. **(C,D)** qRT-PCR **(C)** and Western blot **(D)** analysis of M1 (iNOS) and M2 (Arg-1) macrophage marker expression after FPR1 knockdown or TYROBP overexpression. **(E)** ELISA quantification of pro-inflammatory cytokines (IL-8, CCL5, TNF-α, IL-6) in macrophage supernatants after FPR1 knockdown. **(F)** ELISA quantification of pro-inflammatory cytokines after TYROBP overexpression. **(G)** Western blot analysis of MAPK pathway activation (p-ERK1/2, p-p38) after FPR1 knockdown or TYROBP overexpression. *, *P* < 0.05; **, *P* < 0.01; ***, *P* < 0.001.

Compared to macrophages derived from healthy controls, the mRNA expression levels of FPR1 and TYROBP in peripheral blood macrophages from CRSwNP patients were significantly elevated (*P* < 0.001, Figure 9A), which was consistent with their expression trends in nasal polyp tissues. qRT-PCR analysis confirmed that, compared with the si-NC group, FPR1 mRNA expression was significantly reduced in the FPR1 siRNA transfection group (*P* < 0.001); and compared with the Vector group, TYROBP mRNA expression was significantly increased in the TYROBP-OE group (*P* < 0.001) (Figure 9B). These results indicate that the gene transfection procedures were successful and can be applied in subsequent functional experiments. In addition, the expression of the M1 macrophage marker iNOS and the M2 marker Arg-1 was assessed by qRT-PCR and Western blot. The results showed that compared with the si-NC group, the FPR1 knockdown group exhibited no statistically significant differences in mRNA and protein expression of iNOS (*P* > 0.05), but the mRNA and protein expression of Arg-1 was significantly decreased (*P* < 0.05). Compared with the Vector group, the TYROBP-OE group showed no significant change in iNOS expression (*P* > 0.05), whereas the mRNA and protein expression of Arg-1 was significantly increased (*P* < 0.05) (Figures 9C,D). These findings suggest that both FPR1 and TYROBP specifically regulate macrophage polarization toward the M2 phenotype, with no obvious effect on M1 polarization, which is consistent with the macrophage enrichment conclusion from the bioinformatics analysis. Additionally, ELISA detection of inflammatory factor concentrations in macrophage culture supernatants showed that, compared with the si-NC group, FPR1 knockdown significantly reduced the secretion of pro-inflammatory factors IL-8, CCL5, TNF-α, and IL-6 (*P* < 0.05, Figure 9E); compared with the Vector group, TYROBP overexpression significantly increased the secretion of IL-8, CCL5, TNF-α, and IL-6 (*P* < 0.05, Figure 9F). These results indicate that FPR1 and TYROBP promote the release of pro-inflammatory factors to amplify inflammation, further supporting their roles in the maintenance of chronic inflammation in CRSwNP. Western blot analysis of key molecules in the MAPK pathway revealed that, compared with the si-NC group, FPR1 knockdown significantly reduced the protein levels of p-ERK1/2 and p-p38 in macrophages (*P* < 0.05), while the total protein expression of ERK1/2 and p38 showed no significant change (*P* > 0.05); compared with the Vector group, TYROBP overexpression significantly increased the levels of p-ERK1/2 and p-p38 (*P* < 0.05), with no significant change in the total protein expression of ERK1/2 and p38 (*P* > 0.05) (Figure 9G).

## 4 Discussion

Despite the significant role that immune - related cell death signatures play in the progression of diverse human cancers (Sun et al., 2024), the specific molecular mechanisms and prognostic significance of immune - related PCD genes in CRSwNP remain largely unknown. This study elucidated the crucial role of immune cells in CRSwNP through comprehensive analyses of immune cell expression profiles, differential gene expression, gene co-expression networks, and machine learning-based screening of immune-related genes, culminating in the successful construction of an immune gene-based prognostic model. Our findings revealed that the immune microenvironment of CRSwNP patients exhibits significant immune cell infiltration, particularly characterized by macrophages, mast cells, immature dendritic cells, and neutrophils. Furthermore, key immune-related genes identified through gene expression analysis (including *CD209*, *CYBB*, *FPR1*, *IL2RB*, and *TYROBP*) were not only upregulated in CRSwNP patients but also demonstrated strong correlations with immune cell infiltration, especially macrophages. Drug prediction analysis showed Sulfinpyrazone and Azacyclonol exhibited the highest combined scores with five signature genes, which were promising drug candidates that modulate the activity of these genes. The expression patterns of these genes demonstrated substantial clinical diagnostic value in prognostic assessment and provided novel perspectives for the selection of immunotherapeutic targets.

In recent years, PCD related prospective genes and associated molecular functions have attracted increasing attention in CRSwNP (Cao et al., 2024). Cluster of Differentiation 20 (*CD209*) is a crucial pattern recognition receptor expressed on dendritic cells and has been previously implicated in the regulation of immune responses in chronic airway inflammation (Mulligan et al., 2011). Cytochrome B-245 Beta Chain (*CYBB*), encoding the β subunit of the NADPH oxidase complex, plays a vital role in respiratory burst and pathogen elimination in phagocytes, with research indicating its dysregulation in inflammatory disease (Nambu et al., 2022). Formyl Peptide Receptor 1 (*FPR1*) mediates chemotaxis and inflammatory responses, showing elevated expression patterns in various chronic inflammatory conditions (Yi et al., 2024). Interleukin 2 Receptor Subunit Beta (*IL2RB*), essential for T-cell responses, has been associated with inflammatory disease progression, particularly in sinonasal inflammation (Wu et al., 2024). Transmembrane Immune Signaling Adaptor TYROBP (*TYROBP*), a key adaptor protein in immune cell signaling, has demonstrated significant involvement in myeloid cell activation and inflammation (Zhang et al., 2022). A recent studies highlighting its role in CRSwNP via participating in neutrophil infiltration (Guo et al., 2024). Although these genes have been shown to play a significant role in the progression of inflammatory diseases in humans, there is limited evidence regarding their involvement in CRSwNP. Through current multiple machine learning approaches, we identified these five feature genes including *CD209*, *CYBB*, *FPR1*, *IL2RB* and *TYROBP* as crucial markers. Their elevated expression in CRSwNP samples demonstrated statistical significance, and ROC curve analysis revealed high diagnostic value with consistent performance across both training and validation cohorts. Moreover, the strong associations between immune cell infiltration patterns and disease outcomes in chronic inflammatory conditions has been revealed recent years. Specifically, research has shown that the interaction between tissue-resident macrophages and their associated genetic signatures can serve as robust predictors of disease progression in CRSwNP (Zhu et al., 2022). It has been proved that that M2 macrophage-related gene signature contribute to the development of CRSwNP via immune response (Zhu et al., 2022). Fernandez-Martinez et al. demonstrated that the combination of immune cell profiling and gene expression analysis provides superior prognostic value compared to traditional clinical parameters alone (Fernandez-Martinez et al., 2023). In our clinical sample validation phase, we confirmed the elevated expression of these five genes in CRSwNP patients through qRT-PCR and Western blot analyses. These findings further substantiated the crucial role of macrophages in CRSwNP pathogenesis, suggesting that prognostic models based on these immune-related genes hold promising clinical applications for early diagnosis and personalized treatment strategy development in CRSwNP.

The immune mechanisms of CRSwNP have been a focus of research. Through immune cell expression analysis in this study, both ssGSEA and CIBERSORT algorithms revealed significant differences in the composition of immune cells between the CRSwNP group and the normal group, particularly in the infiltration of macrophages, mast cells, neutrophils, and immature dendritic cells. The macrophages are central to the inflammatory response, exhibiting a dual function of both maintaining tissue homeostasis through phagocytosis and promoting inflammation (Li et al., 2023). In CRSwNP, macrophages often polarize toward an M2 phenotype, secreting cytokines such as IL-10 that support chronic inflammation and tissue remodeling (Wang et al., 2018). Additionally, M2 macrophages can enhance the infiltration of eosinophils, further exacerbating the inflammatory microenvironment in nasal polyps (Liu et al., 2024). Mast cells contribute to both allergic responses and chronic inflammation. Their activation is closely linked to immune evasion and tissue remodeling in CRSwNP (Belsky et al., 2021). Neutrophils contributing to tissue damage through the release of enzymes and inflammatory cytokines (Yu and Kim, 2022). Immature dendritic cells are key in antigen presentation and the activation of T cells, and exacerbate chronic inflammation in CRSwNP (Wang Y. et al., 2024). These immune cells play vital role in the onset and CRSwNP development, and closely associated with chronic inflammatory responses, immune evasion, and nasal polyp formation. In addition to immune cells, immune related pathways are also involved in the process of rhinitis. Zhang et al. demonstrated the inhibition of the PI3K-PKB pathway in rhinitis patients (Zhang et al., 2024). Their findings offer valuable insights into the immunological mechanisms that underpin the efficacy of sublingual immunotherapy. A recent study shows that the immune-associated pathways take part in the progression of CRSwNP, and provided an promising therapeutic option for patients (Bachert et al., 2024). In current WGCNA analysis, we identified a gene module closely associated with macrophages (blue module) that was highly expressed in CRSwNP samples and strongly correlated with immune response and extracellular matrix-related pathways, such as the cytokine-cytokine receptor interaction pathway. These findings suggested that macrophages, by activating specific immune pathways, may play a key role in the chronic inflammation process of CRSwNP.

Through clustering analysis, the current study divided CRSwNP samples into two groups, and identified significant differences between these groups in immune responses and cytokine pathways. Actually, the clustering analysis based on subgroups can lead to a better individualized and targeted management for clinical treatment of CRSwNP. A prior investigation revealed that different CRSwNP clusters characterized by diverse inflammatory mechanisms were strongly associated with various phenotypes (Tomassen et al., 2016). In comparison to relying solely on phenotype information, these clusters offered a more precise account of the underlying inflammatory mechanisms. Isabelle Birs et al. revealed three clusters of asthma with CRSwNP, providing a better individualize treatment for patients combined with CRSwNP and asthma (Birs et al., 2023). By using a single-cell analysis, Ma et al. untangled the complexity of T helper cells in CRSwNP patients. They identified multiple distinct clusters of CD4^+^ T cells, with these clusters showing potential regulatory capabilities (Ma et al., 2021). The distinct immune clusters of CRSwNP may be closely related to the clinical heterogeneity of the disease, such as disease duration and symptom severity. Thus, the clustering finding in current study offered new insights, suggesting that the classification of immune subgroups in CRSwNP could be crucial for the development of personalized treatment strategies. By analyzing different immune clusters, more targeted therapeutic approaches, particularly in immunosuppressive or immunomodulatory treatments, could be provided for patients. However, there were some limitations in current study. Firstly, the relatively small sample size constrained this study. Thus, future large-scale, multi-center validation studies are essential to verify whether these findings can be generalized. Additionally, although our clustering analysis revealed distinct clusters in CRSwNP, the associations between these clusters and clinical phenotypes as well as therapeutic responses require further prospective investigations to validate their utility in guiding personalized treatment strategies.

To further explore the therapeutic potential of these genes, we conducted drug prediction analysis using the DSigDB database, which revealed several promising drug candidates that may modulate the activity of these genes. Among the top predicted drugs, Sulfinpyrazone and Azacyclonol exhibited the highest combined scores, suggesting their potential efficacy in targeting immune-related pathways implicated in CRSwNP. Sulfinpyrazone, primarily known as a uricosuric agent for treating gout and hyperuricemia, has demonstrated anti-inflammatory and immunomodulatory properties in various inflammatory conditions (Halder and Mhaske, 2023). By suppressing neutrophil activation and migration, Sulfinpyrazone might reduce immune cell infiltration in nasal and sinus tissues, thereby alleviating polyp formation and growth. Similarly, Azacyclonol, a psychotropic agent used in the treatment of schizophrenia and anxiety, has potential immunomodulatory effects that could be relevant in human diseases (Karmacharya et al., 2021). While its direct anti-inflammatory role remains underexplored, Azacyclonol may influence neuro-immune interactions, which play a significant role in human diseases (De Bastiani and Klamt, 2019; Xie et al., 2023). The dysregulation of immune cells (e.g., eosinophils, Th2 cells) and inflammatory mediators (e.g., IL-4, IL-5, IL-13) in CRSwNP could be indirectly modulated through Azacyclonol’s effects on neurotransmitter systems and neuropeptide release (e.g., substance P, CGRP). Despite the lack of direct clinical evidence in CRSwNP, both drugs present promising therapeutic avenues. Future research should focus on validating their efficacy and safety in CRSwNP through *in vitro* and *in vivo* studies, as well as clinical trials, particularly for refractory cases or patients with inadequate responses to current therapies.

To further elucidate the functional role of FPR1 and TYROBP in CRSwNP pathogenesis, we conducted *in vitro* experiments using peripheral blood monocyte-derived macrophages from patients and healthy controls. Our findings demonstrated that both FPR1 and TYROBP were significantly upregulated in CRSwNP-derived macrophages, consistent with their expression patterns in nasal polyp tissues. Functional assays revealed that knockdown of FPR1 reduced, while overexpression of TYROBP enhanced, the secretion of pro-inflammatory cytokines (IL-8, CCL5, TNF-α, and IL-6), indicating their roles in amplifying inflammatory responses. Moreover, both genes specifically promoted M2 macrophage polarization, as evidenced by the modulation of Arg-1 expression without affecting iNOS levels. Importantly, we further identified that FPR1 and TYROBP regulate macrophage function through the MAPK signaling pathway, as evidenced by altered phosphorylation levels of ERK1/2 and p38. These results functionally validate the bioinformatics predictions that FPR1 and TYROBP are involved in macrophage infiltration, M2 polarization, and activation of immune-related pathways, thereby contributing to the chronic inflammatory microenvironment in CRSwNP. This provides compelling mechanistic evidence supporting their potential as therapeutic targets in CRSwNP management.

This study has several limitations that should be acknowledged. First, the early functional validation experiments of FPR1 and TYROBP relied on the EoL-1 eosinophilic cell line model. While this model provides insights into certain eosinophil functions, it is a transformed cell line and may not fully recapitulate the behavior of primary eosinophils or other relevant cell types *in vivo*. To address this, we conducted additional experiments using primary cells: human peripheral blood mononuclear cells (PBMCs) were isolated from CRSwNP patients and differentiated into M0 macrophages, followed by FPR1 knockdown and TYROBP overexpression. The results from these primary cell models largely corroborated our initial findings, confirming the roles of FPR1 and TYROBP in regulating macrophage M2 polarization and pro-inflammatory cytokine secretion. However, it remains a limitation that our primary cell model was primarily macrophage-focused and may not fully replicate the complex multicellular interactions within the sinus epithelium or the tissue-resident immune microenvironment of CRSwNP. Second, the relatively limited clinical sample size for both tissue validation and primary cell experiments may restrict the statistical power and generalizability of our conclusions. Future studies should expand the sample size, incorporate more diverse primary cell models (e.g., nasal epithelial cells, tissue-resident immune cells), and conduct multicenter collaborative validation to further elucidate the regulatory mechanisms of these biomarker genes and their potential clinical applications.

In conclusion, we identified five immune-related PCD signature genes, one prognostic model, and two phenotypic clusters for CRSwNP. The potential role of macrophages in the immune-microenvironment of CRSwNP has been raveled. These findings may provide compelling evidence for early diagnosis and personalized therapeutic approaches in CRSwNP management.

## Data Availability

The raw data supporting the conclusions of this article will be made available by the authors, without undue reservation.
