# Supplementary material for: Uncovering the immune-related PCD genes in chronic rhinosinusitis with nasal polyps inflammatory progression: a machine learning and functional validation study
Source: Front Cell Dev Biol. 2026 Mar 2;14:1702613. doi: 10.3389/fcell.2026.1702613 (PMC12989508; doi:10.3389/fcell.2026.1702613)
Supplement: Supplementary file 1 [file DataSheet1.docx]

Supplementary Material

**
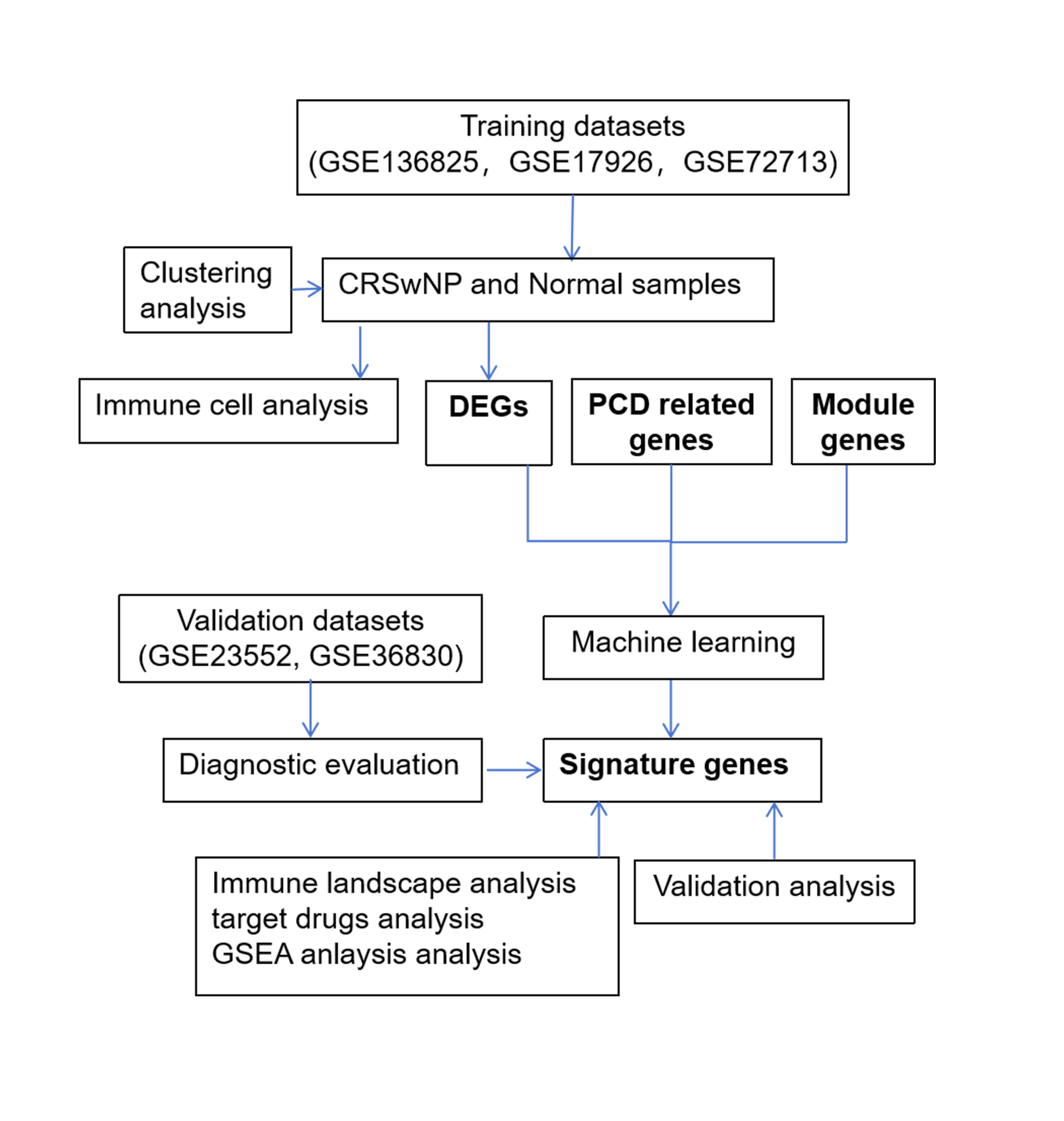
**

**Supplementary Figure 1.** The flowchart for current study.

**
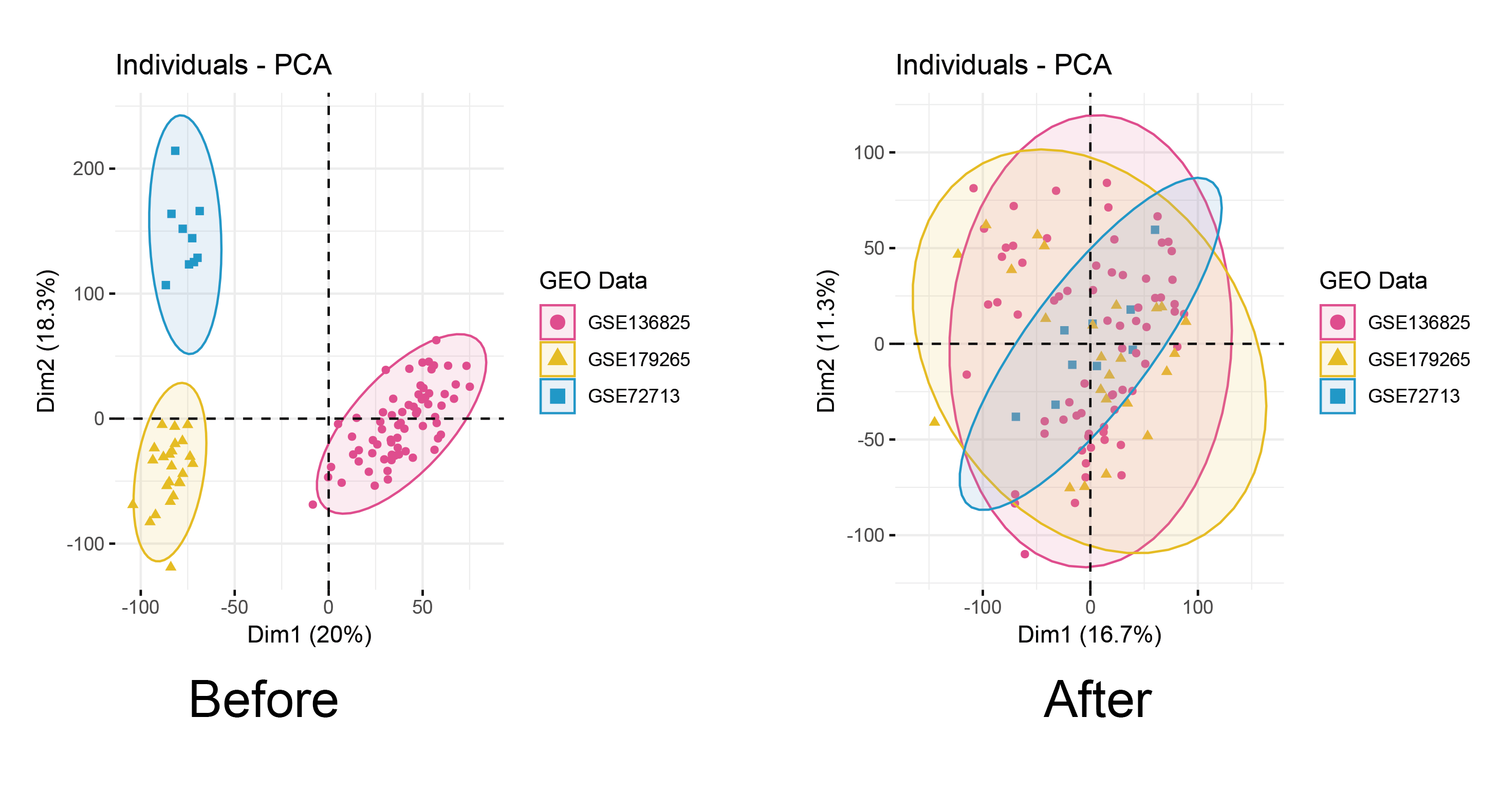
**

**Supplementary Figure 2.** Comparison of PCA distribution plots of expression data before and after batch effect correction.

**
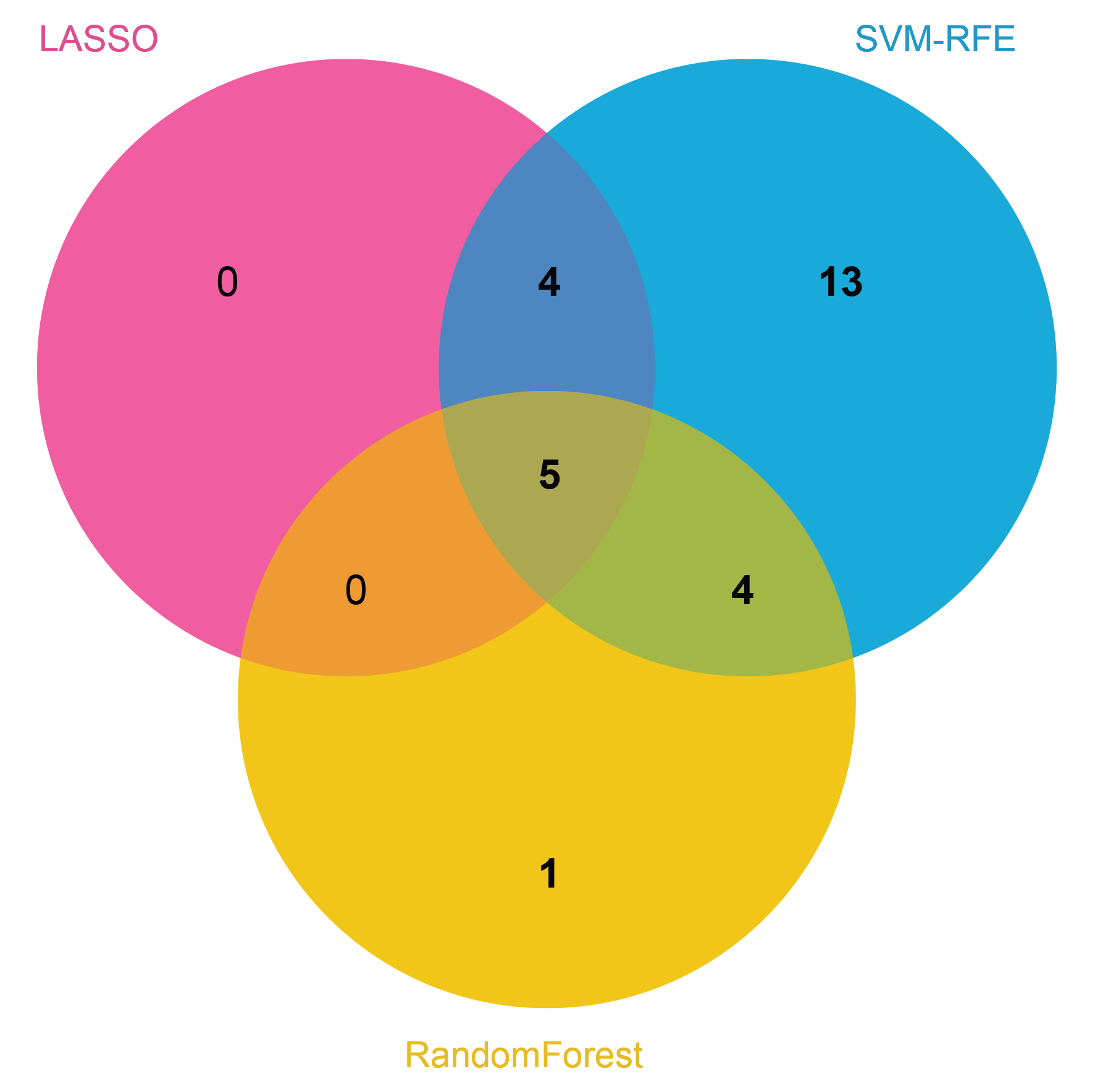
**

**Supplementary Figure 3.** The VENN plot analysis revealed five feature genes for CRSwNP based on the result of four algorithms including LASSO, SVM-RFE and RF.

**
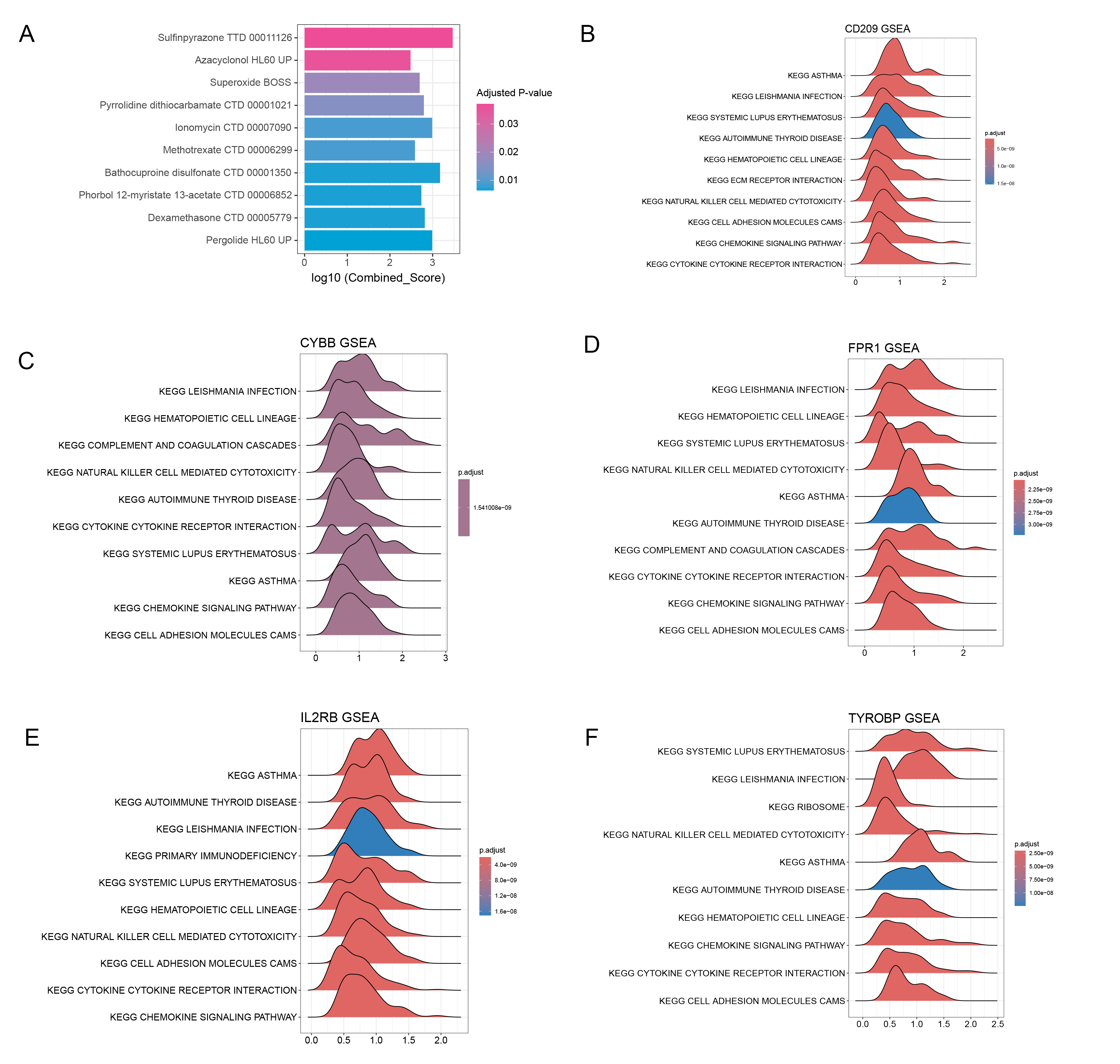
**

**Supplementary Figure 4.** Drug prediction and Gene Set Enrichment Analysis (GSEA) analysis based on signature genes. A, the bar chart showed that TOP 10 drugs closed related to signature genes. B-F, GSEA was conducted on the feature genes of *CD209*, *CYBB*, *FPR1*, *IL2RB* and *TYROBP*, respectively.


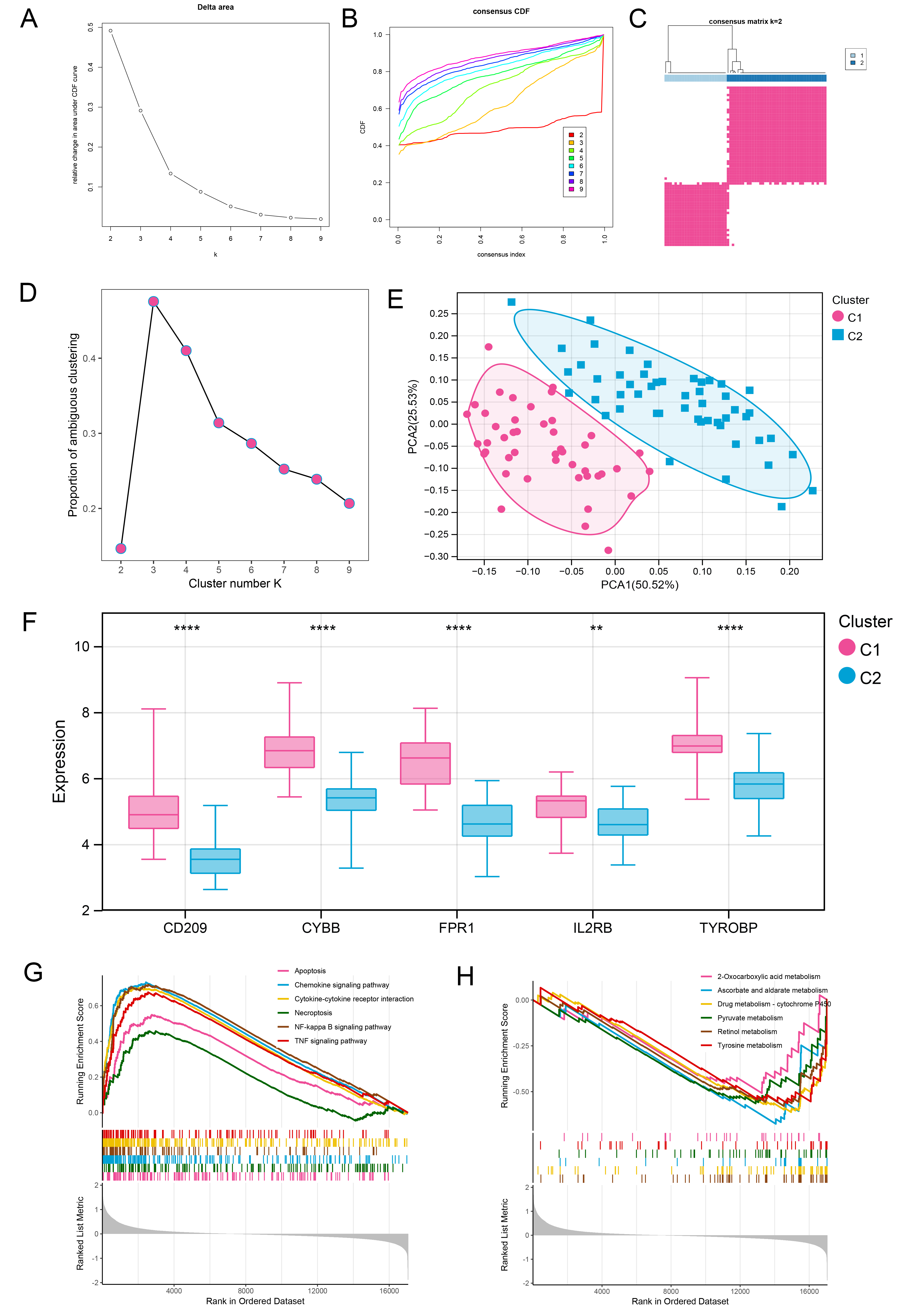


**Supplementary Figure 5.** The clustering analysis of signature genes associated with CRSwNP. A, the clustering analysis based on unsupervised clustering method. B, the result of cumulative distribution function (CDF) analysis. C, minimal area under the curve variation by CDF. D, the result of predictive analysis curve (PAC) analysis. E, the results of principal component analysis (PCA) for clusters. F, the box plot analysis showed that expression of five signature genes in different clusters. G, the TOP 6 up-regulated KEGG pathways between clusters. H, the TOP 6 down-regulated KEGG pathways between clusters. **, *P* < 0.01; ***, *P* < 0.001; ****, *P* < 0.0001.
